# Dawn of Monitoring Regulatory T Cells in (Pre-)clinical Studies: Their Relevance Is Slowly Recognised

**DOI:** 10.3389/fmed.2020.00091

**Published:** 2020-04-02

**Authors:** A. Charlotte M. T. de Wolf, Carla A. Herberts, Marcel H. N. Hoefnagel

**Affiliations:** Medicines Evaluation Board (MEB), Utrecht, Netherlands

**Keywords:** regulatory T cells, immunomonitoring, monoclonal antibodies, JAK inhibitors, registration dossiers, biomarkers, (pre-)clinical study recommendations

## Abstract

Regulatory T cells (Tregs) have a prominent role in the control of immune homeostasis. Pharmacological impact on their activity or balance with effector T cells could contribute to (impaired) clinical responses or adverse events. Monitoring treatment-related effects on T cell subsets may therefore be part of (pre-)clinical studies for medicinal products. However, the extent of immune monitoring performed in studies for marketing authorisation and the degree of correspondence with data available in the public domain is not known. We evaluated the presence of T cell immunomonitoring in 46 registration dossiers of monoclonal antibodies indicated for immune-related disorders and published scientific papers. We found that the depth of Treg analysis in registration dossiers was rather small. Nevertheless, data on treatment-related Treg effects are available in public academia-driven studies (post-registration) and suggest that Tregs may act as a biomarker for clinical responses. However, public data are fragmented and obtained with heterogeneity of experimental approaches from a diversity of species and tissues. To reveal the potential added value of T cell (and particular Treg) evaluation in (pre-)clinical studies, more cell-specific data should be acquired, at least for medicinal products with an immunomodulatory mechanism. Therefore, extensive analysis of T cell subset contribution to clinical responses and the relevance of treatment-induced changes in their levels is needed. Preferably, industry and academia should work together to obtain these data in a standardised manner and to enrich our knowledge about T cell activity in disease pathogenesis and therapies. This will ultimately elucidate the necessity of T cell subset monitoring in the therapeutic benefit-risk assessment.

## Introduction

The mammalian immune system is indispensable for the protection against a broad range of pathogens. For this, immune cells should be able to differentiate between (pathogenic) non-self and self. In addition, immune responses should be fine-tuned to demarcate the localisation and extent of an inflammatory reaction. Preservation of self-tolerance and immune homeostasis is mediated by various immunosuppressive mechanisms, including regulatory T cells (Tregs) (1). These suppressor cells appear to be specifically equipped to control the activation of other immune cells (2).

Human Tregs can be classified in different subtypes. The major subtype consists of the classical CD4^+^ Tregs that are either differentiated in the thymus (also known as natural Tregs) or peripherally induced from conventional (effector) CD4^+^ T cells (3, 4). Classical Tregs highly express CD25 [i.e., interleukin (IL)-2α receptor] and cytotoxic T lymphocyte-associated antigen-4 (CTLA-4) (5–7). These surface markers, together with the transcription factor forkhead box protein 3 (FoxP3), have essential roles in Treg-mediated suppressive functionality (8–12). Non-classical Tregs include FoxP3^−^ Tr1 and T helper (Th)3 cells. These types are depending on IL-10 and tumour growth factor-β production for their suppressive activity (13, 14). Also γδ T cell and CD8^+^ T cell populations contain suppressive subsets, but their specific roles in regulating the immune system have yet to be identified (15–19).

The negative regulation of an immune response as mediated by Tregs is essential to prevent auto-immune and allergic disorders. On the other hand, this suppressive activity may prevent pathogen clearance during infections and hinder effective immune responses against (mutated) self-antigens in cancer (20). Therefore, in diseases where the balance between immune activation and suppression is skewed, Tregs could be attractive pharmacological targets (21, 22). For Th1- and Th17-dominated auto-immune disorders and Th2-dominated allergies, a therapy increasing Treg suppressive activity is sought (21, 23, 24). In contrast, for malignant diseases reversing an immunosuppressive tumour micro-environment by reducing Treg functionality would be the goal of treatment (21, 24–26). However, targeting Tregs *in vivo* is challenging, because a single (surface) marker with high specificity and selectivity for Tregs is still lacking (25). In addition, interfering with Treg numbers and/or functionality may also increase the risk for (auto-)immune-related adverse events (8). Examples are auto-immune enterocolitis and myocarditis following treatment with immune checkpoint inhibitors such as anti-CTLA-4 and anti-programmed cell death-1 (PD-1) (27–33). But also therapies against auto-immune disorders, for example tumour necrosis factor (TNF) inhibitors, have been reported to result in paradoxical immune-related inflammation (34).

Given the role of Tregs in (maintenance of) the immune balance, inclusion of these cells in the investigation of treatment effects on T cell subsets would be expected to be part of the (clinical) development program of medicinal products, at least for therapies targeting the immune system. Comprehensive overviews of immunomodulatory therapy-related effects on the balance between effector and regulatory T cells are available, for example for arthritis and solid organ transplantation (21, 35, 36). They show that general immunosuppressive drugs (such as corticosteroids), which target intracellular signalling pathways, do not only affect conventional T cell activation, but may also affect Treg activity. However, the sensitivity to the pathway-suppressive effects of these products differs between effector and regulatory T cells, and this difference determines whether immunomodulatory products will inhibit or stimulate immune cell activity. Differences in inhibition sensitivity of shared intracellular pathways are also apparent for more selective immunomodulating drug products. For example, blocking TNF has an effect on both TNF receptor-expressing effector T cells and Tregs, although it appears that positive clinical responses in several auto-immune disorders are the result of a greater inhibition of the effector than the regulatory cells (37).

Medicinal products may also disturb the balance between effector and regulatory T cells or the total T cell population more indirectly or even unintendedly (i.e., off-target effects). For example, monoclonal antibody (mAb)-mediated apoptosis results in the tumour tissue infiltration of immune cells, including Tregs. These Tregs can negatively influence the cytotoxic potential of effector cells, which could result in reduced efficacy. Therefore, immunomonitoring in (pre-)clinical studies is a useful tool to elucidate unintended treatment effects (and potential underlying mechanisms) caused by disturbance of the immune balance. In addition, immunomonitoring can provide more insight in the role of specific immune cells in the disease pathophysiology and thereby contribute to the identification of biomarkers predictive for the clinical response (38).

Given the potential clinical impact of Treg modulation, appropriate monitoring of treatment-induced effects on Treg frequency, phenotype and function would be required. We questioned whether Tregs have been investigated in (pre-)clinical studies to support a marketing authorisation application (MAA). Therefore, we surveyed if and when T cells, and Tregs in particular, were evaluated in these studies and whether the data in the registration dossiers corresponded to the available data in the public domain. There are multiple immunomodulatory therapies registered in the EU. We have chosen to restrict the sample size of registration dossiers to MAAs for approved mAb products based on the assumption that for mAbs immunomonitoring studies most frequently have been performed. After all, the majority is indicated for immune system-related disorders. In addition, we assessed T cell monitoring for a few tyrosine kinases inhibitors known to specifically target cytokine signalling pathways in T cells. We conclude this review with our perspective on the value of Treg monitoring and recommendations for their evaluation in (pre-)clinical studies.

## Search for Immunomonitoring Data

### Selection of Monoclonal Antibodies

We have evaluated the presence of data on T cell immunomonitoring (with the focus on Tregs) reported in published literature and in registration dossiers for MAA. We included all mAb products used as anti-neoplastic agents (anatomical therapeutic chemical classification code L01XC) or as selective immune inhibitor in the context of auto-immunity (L04AA, AB and AC) or asthma (R03DX), which have been EU-registered between 2006 and the first half year of 2019. Products that have been authorised and subsequently withdrawn in this time frame (for commercial, insufficient supply or unfavourable benefit-risk reasons) have been included. Biosimilars were excluded from our evaluation, because it was not expected that information on Tregs would be included in these dossiers (39). In total, 46 monoclonal antibodies were considered eligible.

### Selection of Publications and Registration Dossier Reports

**Figure 1:**
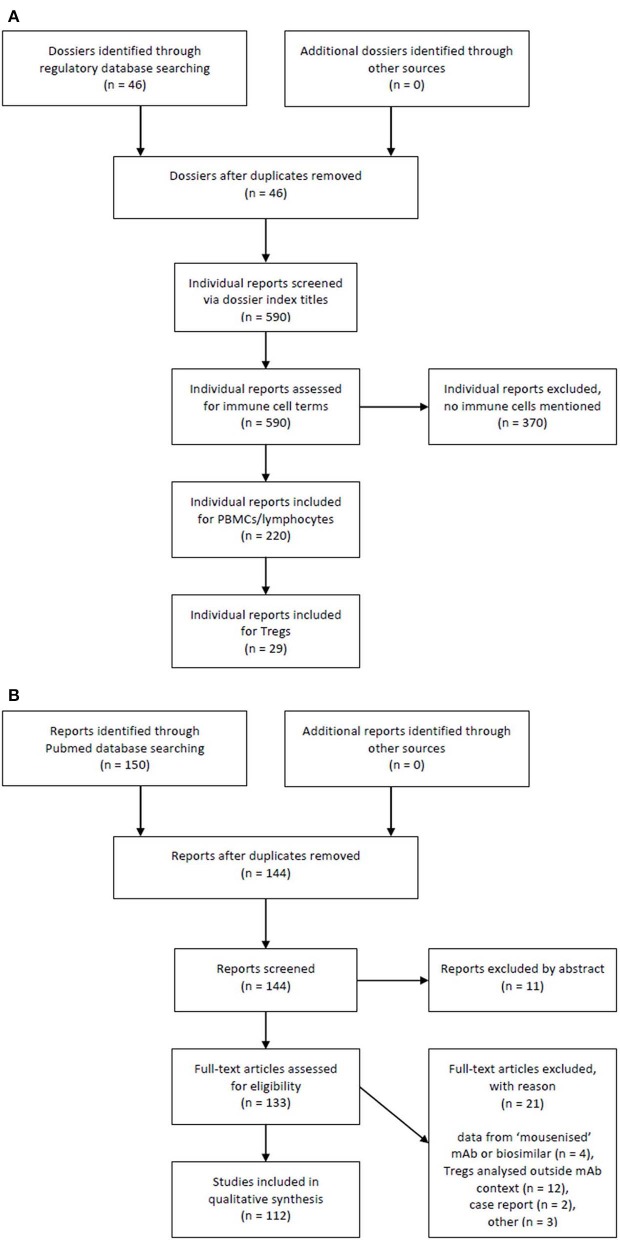
Flow chart of registration dossiers and literature reports selection process. **(A)** Registration dossier search and **(B)** literature search.

As far as applicable, this study followed the recommendations of PRISMA in conducting and reporting a systemic review. Registration dossier search is summarised in Figure 1A, literature search is summarised in Figure 1B.

We searched in both registration dossiers [common technical documents, CTDs, required to apply for regulatory approval of a new medicinal product (40)] and PubMed literature for Treg-related keywords (including: regulatory T cell, suppressor T cell, Treg, FoxP3, CD25, mAb generic, and trade names). Because the Treg field is relatively new and the extent of T cell monitoring in dossiers of mAb products was not known, we decided to search more generally for lymphocyte and T cell populations in the CTDs, but with a focus on Tregs. Therefore, we also included keywords related to the whole T cell population (such as: lymphocyte, T cell, CD4, and CD8). When lymphocytes or T cells were mentioned in an individual study report, we also searched with the Treg-related keywords. Our main focus was on CD4^+^ FoxP3^+^ T cells, because these are the major Tregs in the immune system. However, other (non-classical) suppressor T cells—when mentioned in reports—were also taken along.

To explore lymphocyte and T cell immunomonitoring in registration dossiers, we searched the pre-clinical and clinical sections of the CTD (pre-clinical module 4 and clinical module 5, respectively) for each individual monoclonal antibody. Most study reports containing immunomonitoring results were found in the sections about pre-clinical pharmacology (module 4.2.1), toxicology (module 4.2.3), clinical pharmacokinetics (module 5.3.3), efficacy and safety (module 5.3.5).

## Results

### Evaluation of Immunomonitoring Data Availability

We investigated whether potential mAb treatment-related effects on Tregs reported in published studies are also reported as individual studies presented in registration dossiers. Table 1 describes the immunomonitoring parameters used to determine potential effects of mAb products on lymphocytes, T cell subsets or specifically Tregs in registration dossier reports. In this table, no individual mAb products have been indicated for confidentiality reasons. The effects of the individual mAb products on Treg frequency, phenotype, and function as found in literature are described in Tables 2–4. Table 5 represents all mAb products for which no scientific literature was publicly available. Product-related effects were measured either pre-clinically (*in vitro* or *in vivo* in animal models) or clinically (human healthy donors and patients), the latter further subdivided in systemic (in peripheral blood) and local (at the tumour site or in inflamed tissue) effects. Potential (absence of) associations between clinical results and the presence or activity of Tregs prior to or during/after treatment are included.

**Table 1:** Overview of lymphocytic parameters evaluated in mAb registration dossiers.

***In vivo* target group (# of mAb dossiers evaluated)**	**Parameters determined on lymphocytes or T cells (# of mAb dossiers)**	**Treg identification markers^**a**^**	**Parameters determined on Treg frequency and phenotype (# of mAb dossiers)**	**Parameters determined on Treg functionality (# of mAb dossiers)**	**Clinical associations (# of mAb dossiers)**
Target highly relevant for Tregs (7)	*Pre-clinical* •Amount of total T cell population (1)^b^ •Amount of (naïve/memory) CD4^+^ and/or CD8^+^ T cells (5) •Activation level of CD4^+^ and/or CD8^+^ T cells (2) •Cytokine production of CD4^+^ and/or CD8^+^ T cells (1) •Functionality of CD4^+^ and/or CD8^+^ T cells (1)	*Pre-clinical* CD4, CD25, CD127; CD4, CD25, FoxP3; CD8, CD25, FoxP3; Unknown	*Pre-clinical* •Binding potential to T suppressor cells (1) •Amount of (mAb-target^+^) CD4^+^ suppressor cells (3) •Amount of CD8^+^ suppressor cells (2)	*Pre-clinical* n.a.	*Prognostic* •Association between baseline FoxP3 expression of T suppressor cells and clinical benefit (1) *Predictive* •Association between amount of CD4^+^ or CD8^+^ T cells and clinical response rate (1)
	*Clinical* •Amount of total lymphocyte population (1) •Amount of total T cell population (1)^b^ •Amount of (naïve/memory) CD4^+^ and/or CD8^+^ T cells (6)^c^ •Activation level of CD4^+^ and/or CD8^+^ T cells (1)	*Clinical* CD4, CD25, FoxP3	*Clinical* •Amount of CD4^+^ suppressor cells (4)^c^	*Clinical* n.a.	
Target relevant for the T cell population (28)^d^	*Pre-clinical* •Viability of total lymphocyte or PBMC population (2) •Amount of total lymphocyte population (7)^c^ •Amount of total T cell population (1)^b^ •Amount of (naïve/memory) CD4^+^ and/or CD8^+^ T cells (14) •Activation level of CD4^+^ and/or CD8^+^ T cells (4) •Cytokine production of PBMC population (1) •Cytokine production of CD4^+^ and/or CD8^+^ T cells (2) •Functionality of total T cell population (1) •Functionality of CD8^+^ T cells (3)	*Pre-clinical* FoxP3; CD4, CD25; CD4, FoxP3; CD4, CD25, CD127; Unknown	*Pre-clinical* •Binding potential to CD45RO^+^ CD4^+^ suppressor cells (1) •Density of tumour-infiltrating suppressor cells (1) •Amount of T suppressor cells (2)^c^	*Pre-clinical* •Suppressive capacity of mAb target^+^ CD4^+^ suppressor cells (1)	*Predictive* •Association between amount of CD4^+^ suppressor cells and clinical response (1)
	*Clinical* •Amount of total lymphocyte population (12)^c^ •Amount of total T cell population (4) •Amount of (naïve/memory) CD4^+^ and/or CD8^+^ T cells (9) •Amount of CD4^+^ T cell subsets (2) •Activation level of CD4^+^ and/or CD8^+^ T cells (3) •CD4^+^ T cell: CD8^+^ T cell ratio (2) •Cytokine production of CD4^+^ T cells (1)	*Clinical* CD4, CD25, FoxP3; CD4, CD25, CD127, FoxP3; Unknown	*Clinical* •Amount of T suppressor cells (2) •Amount of CD4^+^ suppressor cells (3) •Amount of CD8^+^ suppressor cells (1) •CD8^+^ T cell : CD4^+^ suppressor cell ratio (1)	*Clinical* n.a.	
Target not relevant for T cells (11)	*Pre-clinical* •Amount of total lymphocyte population (5)^c^ •Amount of total T cell population (4)^b, c^ •Amount of CD4^+^ and/or CD8^+^ cells (7)^c^	*Pre-clinical* n.a.	*Pre-clinical* n.a.	*Pre-clinical* n.a.	n.a.
	*Clinical* •Amount of total lymphocyte population (6) •Amount of total T cell population (2)^b, c^ •Amount of CD4^+^ and/or CD8^+^ cells (4) •CD4^+^ T cell : CD8^+^ T cell ratio (1)	*Clinical* n.a.	*Clinical* n.a.	*Clinical* n.a.	

a *Summary of phenotypic markers used in the studies (in different combinations) to identify cells that are -according to the company- regulatory cells*.

b *For these mAb products, no T cell subset analysis was performed*.

c *For one or more mAb products, individual, or summarising results were not reported*.

d *For three mAb products, treatment-related effects on lymphocytes (sub)populations were not determined*.

*n.a., not available; PBMC, peripheral blood mononuclear cell*.

To gain better insight in the type of products for which T cell monitoring was available, we divided the mAb products in three groups, based on the relevance of the product's pharmacological target for T cell (subset) function and survival:

1. mAb target is highly relevant for Tregs, i.e., the target is constitutively expressed on most Tregs (e.g., CTLA-4);
2. mAb target is -expected to be- relevant for the T cell population, i.e., the target is expressed on specific T cell and Treg subsets (e.g., α4 integrins) or the mAb product has a more indirect effect on Treg activity, when the target is involved in the balance between T cell subsets (e.g., IL-6 and IL-17A pathways);
3. mAb target is not directly relevant for T cells, i.e., the target is not involved in T cell functionality (e.g., CD20, which is expressed on B cells).

### T Cell Immunomonitoring Data in mAb Registration Dossiers

For the majority of registration dossiers of mAbs targeting Tregs or other T cell subsets, only absolute and relative counts of lymphocytes, lymphocyte subsets (i.e., T and B cells, in some cases also natural killer cells) or T cell subsets (CD4^+^ and CD8^+^) were determined clinically and pre-clinically (Table 1). T cell functionality testing (i.e., proliferative or cytotoxic capacity) was limited to pre-clinical studies, whereas further differentiation of T cell subsets (such as naïve/memory state) and determination of the CD4^+^ to CD8^+^ ratio was primarily found in clinical reports. Most data were derived from samples evaluated via clinical haematology, flow cytometry or immunohistochemistry. In most cases, however, no summarising data or concluding remarks (such as clinical significance) concerning the treatment effects on T cell frequency and functionality were provided.

### Comparison of Treg Immunomonitoring Between Literature and mAb Registration Dossiers

#### Targets With High Relevance for Regulatory T Cells

Seven mAb products were classified as affecting targets (here: cell surface receptors) essential for Treg function or survival. In literature, treatment-related effects on frequency and phenotype (other than identity markers such as CD25 and FoxP3) were studied for all these mAb products, whereas effects on suppressive function were evaluated for four mAbs (Table 2). Nevertheless, high variability between mAb products existed in the number of available studies (most on products targeting CTLA-4 and PD-1) and the level of Treg analysis per study. For the majority of the publications, identification of Tregs within the T cell population was based on several markers (mainly a combination of CD25, CD127, and/or FoxP3) to exclude activated effector T cells as much as possible. In four of the seven mAb registration dossiers, effects on the frequency of Tregs (defined as CD4^+^/CD8^+^ CD25^+^ FoxP3^+^ in most studies) were taken into account (Table 1), although actual results were not always reported. Next to frequency, Treg functionality is an important determinant of the degree of immune suppression and thus requires evaluation. But in none of the dossiers Treg functionality was determined.

**Table 2:** Overview of published studies for mAbs with a target highly relevant for Tregs.

***In vivo* target and monoclonal antibody (trade mark, year of EU registration)**	**Treg identification markers^**a**^**	**Effect of mAb on Treg frequency or phenotype**	**Effect of mAb on Treg functionality**	**Clinical associations**	**Key references**
CCR4 Mogamulizumab (Poteligeo, 2018)	Pre-clinical *Peripheral blood* CD4, CD45RA, FoxP3 Clinical *Peripheral blood* CD4, CD25, FoxP3; CD4, CD25, CD127, FoxP3; CD4, CD45RA, FoxP3 *Tumour site* FoxP3; CD4, FoxP3; CD4, CD45RA, FoxP3	Pre-clinical *Peripheral blood* •↓ % Tregs (mainly eTregs) Clinical *Peripheral blood* •↓ number of Tregs (mainly eTregs) •↓ % Tregs (mainly eTregs) •↓ FoxP3 mRNA expression *Tumour site* •↓ % Tregs (mainly eTregs) •↓ FoxP3 expression	n.a.	Predictive *Peripheral blood* •↓ % Tregs is associated with induction of skin-related adverse events •Association between changes in % eTregs and occurrence of tumour relapse vs. adverse events •No relation between changes in % (effector) Tregs and extent of clinical response	(41–51)
CD3 and CD19 Blinatumomab (Blincyto, 2015)	Pre-clinical *In vitro* CD4, CD25, CD127 Clinical *Peripheral blood* CD4, CD25, FoxP3	Pre-clinical *In vitro* •↑ CD25, CD69 and PD-1 protein expression (HC) •↑ IL-10 and ↓ IFN-γ and TNF-α production (HC) Clinical n.a.	Pre-clinical *In vitro* •↑ Treg-mediated suppression of T effector cell proliferation and lytic capacity (HC) Clinical n.a.	Predictive *In vitro* •Depletion of Tregs in non-responders can restore the T effector cell proliferation*Peripheral blood* •↑ % Tregs is associated with decreased therapy responsiveness	(52)
CD3 and EpCAM Catumaxomab (Removab, 2009)*	Pre-clinical *In vitro* CD4, FoxP3 Clinical *Peripheral blood* CD4, FoxP3	Pre-clinical *In vitro* •↑ % Tregs (mainly FoxP3^high^ CD45RA^−^ CD73^+^ subpopulation)Clinical *Peripheral blood* •≈ % Tregs •No expression of FoxP3 protein in CD4^+^ cells when *ex vivo* restimulated with EpCAM peptides	n.a.	n.a.	(53, 54)
CTLA-4 Ipilimumab (Yervoy, 2011)	Pre-clinical *In vitro* CD4, CD25, FoxP3 *Animal model* CD4, CD25, CD127, FoxP3	Pre-clinical *In vitro* •Treg lysis by mAb-activated CD16^+^ monocytes *Animal model* •↑ number of Helios^+^ Tregs (hM)	Pre-clinical *In vitro* •Treg-mediated suppressive capacity is not affected *Animal model* n.a.	Prognostic *Animal model* •↓ local Treg : Teff ratio is associated with weight loss (hM) *Peripheral blood* •Positive correlation between	(30, 33, 55–86)
	Clinical *Peripheral blood* CD4, CD25, CD39; CD4, CD25, FoxP3; CD4, CD25, CD127, FoxP3 *Tumour site* CD4, FoxP3	Clinical *Peripheral blood*•↑ or ≈ number of Tregs •↓ number of CD62L^+^ Tregs, followed by ↑ compared to baseline level •↑, ≈ or ↓ % Tregs •↑ % CD39^+^ and/or Helios^+^ Tregs •↑ % Tregs, followed by ↓ compared to baseline level •↑ CD4^+^ ICOS^+^ T cell : Treg ratio •↓ CD8^+^ T cell : Treg ratio •↑ Ki67 protein expression in CD45^+^ Tregs •↑ FoxP3 mRNA expression in Tregs •↓ FoxP3 mRNA expression in PBMCs, followed by ↑ compared to baseline level (rebound)*Tumour site* •↑ % Tregs compared to blood •↓ % Tregs •↑ CD4^+^ ICOS^+^ T cell : Treg ratio •≈ CD8^+^ T cell : Treg ratio •≈ Ki67^+^ effector T cell : Treg ratio •↓ CD8^+^ T cell : Treg ratio compared to blood •↓ FoxP3 mRNA expression	Clinical *Peripheral blood*•Treg-mediated suppressive capacity is not affected *Tumour site* n.a.	pre-treatment number of CTLA^−^ Tregs (but not CTLA-4^+^ Tregs) and overall survival •Positive association between pre-treatment % of CD39^+^ Tregs and relapse-free survival *Tumour site* •↑ number of FoxP3^+^ cells in pre-treatment metastatic tumour lesions is associated with clinical response (a.o., overall survival) •↑ % Tregs in pre-treatment tumour lesions is associated with clinical activity Predictive *Peripheral blood* •No difference in number of Tregs between patients with and without immune-related adverse events •↓ number of Tregs is associated with local and systemic clinical benefit •↑ % Tregs is associated with improved progression-free survival •↑ % Treg suppressive function is associated with decreased progression-free survival •↑ % Helios^+^ and/or HLA-DR^+^ Tregs is associated with decreased clinical response •↓ % Tregs is associated with overall survival •No association between changes in Treg frequency and function •No association between changes in % Tregs and clinical response (a.o., overall survival) *Tumour site* •Inverse correlation between number of intra-tumoural Tregs and the degree of tumour necrosis after treatment •↓ % Tregs in post-treatment tumour lesions from responders compared to non-responders •No association between intra-tumoural FoxP3 protein expression and clinical response	
PD-1 Nivolumab (Opdivo, 2015)	Pre-clinical *In vitro* CD4, CD25; CD4, CD25, FoxP3 *Animal model* CD4, CD25, FoxP3 Clinical *Peripheral blood* CD4, CD25*Tumour site* CD4, CD25	Pre-clinical *In vitro* •↓ % (Ki67^+^) Tregs *Animal model* •≈ number of Tregs (hM) •≈ CD8^+^ T cell : Treg ratio (hM) Clinical *Peripheral blood* •↑ % Tregs •↑ % CCR4^+^ Tregs •≈ % TCRαβ^+^ CD45RO^+^ Tregs *Tumour site* n.a.	Pre-clinical *In vitro* •↓ Treg-mediated suppressive capacity*Animal model* n.a.Clinical n.a.	Prognostic *Peripheral blood* •↓ pre-treatment % Tregs is associated with non-relapsing •↓ pre-treatment % PD-1^+^ Tregs is associated with positive clinical response*Tumour site* •↑ pre-treatment FoxP3 (mRNA) expression is associated with diminished survival ↓ pre-treatment % PD-L1^+^ Tregs is associated with diminished clinical outcome	(87–94)
Pembrolizumab (Keytruda, 2015)	Pre-clinical *In vitro* CD4, CD25 Clinical *Peripheral blood* CD4, CD25, CD127, FoxP3 *Tumour site* CD4, CD25, CD127	Pre-clinical *In vitro* •≈ CD15s, CTLA-4, FoxP3, Helios, Ki67 and LAP protein expression on/in TregsClinical*Peripheral blood* •≈ % Tregs •≈ % CD45^+^ Tregs •↑ CD8^+^ T cell : Treg ratio*Tumour site* •≈ % Tregs •≈ % CD45^+^ Tregs	Pre-clinical *In vitro*•Treg-mediated suppressive capacity is not affected (HC) Clinical n.a.	Prognostic *Tumour site* •↓ pre-treatment % PD-L1^+^ Tregs is associated with diminished clinical outcome	(68, 87, 95–97)

a *Summary of phenotypic markers used in the studies (in different combinations) to identify cells that are -according to the authors- regulatory cells*.

* *This mAb is now withdrawn from use in the European Union*.

*↓, decreased level (compared to baseline or control); ↑, increase (compared to baseline or control); ≈, similar level (compared to baseline or control); CCR4, CC-chemokine receptor 4; CD15s, Sialyl Lewis x; EpCAM, epithelial cell adhesion molecule; eTreg, effector (activated) regulatory T cell; HC, healthy controls; HLA-DR, one of the human MHC class II molecules; hM, humanised mice; ICOS, inducible T cell co-stimulator; IFN, interferon; Ki67, intracellular marker for proliferation; LAP, latency-associated peptide; n.a., not available; PBMC, peripheral blood mononuclear cell*.

In both publications and registration dossier studies, mAb-related effects on Tregs were found. However, comparing these sources elucidated a clear discrepancy. For most mAb products, the public domain contained more studies and within these studies, Tregs were analysed more extensively than in registration dossiers. For example, activation markers (such as Ki67 and CD69), phenotypic and functionality-related markers (e.g., Helios, CD39, PD-1, CTLA-4, cytokines such as IL-10 and the combination of CD45RA^−^ with FoxP3^++^) or actual suppressive activity were monitored for all mAb products in published studies, except cemiplimab. In contrast, in only one registration dossier, two of such markers (i.e., CTLA-4 and CCR4, thus directly related to the mAb target) were taken into account. Analysis of surface markers next to standard Treg identity indicators could be a useful surrogate for Treg activity when actual functionality assays cannot be performed [see section Recommendations for (Pre-)clinical Studies].

In several public studies, Treg (subset) frequency could be used to differentiate responders from non-responders or to predict the clinical response prior to treatment. A decrease in local or systemic Treg frequency was associated with a better (anti-tumour) treatment response (52, 55–59). A high Treg frequency at baseline was associated with either better or worse clinical outcome, depending on the evaluated Treg phenotype (60, 61, 87–89). These studies indicate that Tregs may be assigned as potential biomarker for disease activity or clinical outcome [see section Value of Treg Monitoring]. Because Treg data would be available much quicker than, for example, long-term clinical responses such as progression-free survival, it could be worthwhile to investigate applicability of such biomarker in product-specific studies (as surrogate clinical end point) (144). Nevertheless, this probably requires more (pre-)clinical experience than available at the time of MAA.

#### Targets With Relevance for Tregs or the Balance Between T Cell Subsets

Most evaluated mAb products [i.e., (28)] were designed to target cell surface receptors or cytokines that have a role in the physiology of T cells. All targets (or their receptors) were known to be expressed on Treg subsets or were earlier defined as involved in maintaining the delicate balance between effector and regulatory T cells.

In general, the number of published studies (Table 3) was related to the level of Treg analysis in the registration dossiers (Table 1), except for Treg functionality. However, where Treg identification in literature was based on a combination of several phenotypic markers (e.g., CD25 and CD127 next to CD4), most dossier reports defined Tregs solely on one marker (if defined at all). Therefore, the latter may have measured therapeutic effects on a mix of Tregs and activated effector T cells, which interferes with correct interpretation of the data [see section Recommendations for (Pre-)clinical Studies]. It was also noted that changes in Treg phenotype (e.g., activation) were analysed more in-depth in public literature compared to registration dossiers.

**Table 3:** Overview of published studies for mAbs with a target relevant for the T cell population.

***In vivo* target and monoclonal antibody (trade mark, year of EU registration)**	**Treg identification markers^**a**^**	**Effect of mAb on Treg frequency or phenotype**	**Effect of mAb on Treg functionality**	**Clinical associations**	**Key references**
α4 subunit of integrins Natalizumab (Tysabri, 2006)	Pre-clinical *In vitro* CD4, CD25, FoxP3 Clinical *Peripheral blood* CD4, CD25, FoxP3;CD8, CD28	Pre-clinical *In vitro* •≈ % Tregs Clinical *Peripheral blood* •↑ % Tregs when *ex vivo* restimulated with auto-antigen •↑, ≈ or ↓ % Tregs •≈ % CTLA-4^+^ Tregs •↓ % Helios^+^ Tregs (compared to HC) •≈ FoxP3 mRNA expression in Tregs •↓ FoxP3 mRNA expression in memory CD49d^+^ Tregs •↓ CD49d protein expression on Tregs •↑ CD49d^+^ Th1 cell : CD49d^+^ Treg ratio •↑ CD49d^+^ Th17 cell : CD49d^+^ Treg ratio	Pre-clinical *In vitro* •Treg-mediated suppressive capacity is not affected (HC) •α4^+^ β7^+^ Treg-mediated suppressive capacity is not affected (HC)Clinical*Peripheral blood* •Treg-mediated suppressive capacity is not affected •Migratory capacity of Tregs not affected	Predictive *Peripheral blood* •↑ CD49d^+^ Th1 cell : CD49d^+^ Treg ratio or CD49d^+^ Th17 cell : CD49d^+^ Treg ratio associated with relapse •Negative correlation between IL-10 concentration and disease progression	(98–108)
	*Tissue* FoxP3	*Tissue* •≈ number of Tregs •≈ CD4^+^ T cell : Treg ratio •≈ CD8^+^ T cell : Treg ratio	*Tissue* n.a.		
α4β7 integrin Vedolizumab (Entyvio, 2014)	Pre-clinical *In vitro* CD4, CD25, CD127, FoxP3 *Animal model* CD4, CD25, CD127 Clinical *Peripheral blood* CD4, FoxP3	Pre-clinical *In vitro* n.a. *Animal model* •Treg homing from blood to tissue (hM) Clinical *Peripheral blood* •↑ % Tregs •↓ CD4^+^ FoxP3^−^ cell : Treg ratio	Pre-clinical *In vitro* •α4^+^ β7^+^ Treg-mediated suppressive capacity is not affected (HC) *Animal model* n.a. Clinical n.a.	n.a.	(102, 109)
C5 Eculizumab (Soliris, 2007)	Pre-clinical n.a. Clinical *Peripheral blood* CD4, CD25, FoxP3	Pre-clinical n.a. Clinical *Peripheral blood* •≈ number of Tregs •≈ number of CXCR4^+^ Tregs	Pre-clinical n.a. Clinical *Peripheral blood* •Treg-mediated suppressive capacity is not affected	n.a.	(110)
CD30 Brentuximab vedotin (Adcetris, 2012)	Pre-clinical n.a. Clinical *Peripheral blood* CD4, CD25, CD127	Pre-clinical n.a. Clinical *Peripheral blood* •↓ % CCR4^+^ Tregs	n.a.	Prognostic *Peripheral blood* •No correlation between pre-treatment % CD30^+^ Tregs and clinical response	(91, 111)^b^
CD38 Daratumumab (Darzalex, 2016)	Pre-clinical n.a. Clinical *Peripheral blood* CD4, CD25, CD127	Pre-clinical n.a. Clinical *Peripheral blood* •↓ number of Tregs •↓ number of CD38^+^ Tregs •↓ % CD38^+^ Tregs •↑ CD8^+^ T cell : Treg ratio	n.a.	Prognostic *Peripheral blood* •Positive correlation between pre-treatment number of CD38^+^ Tregs (but not total Tregs) and extent of the responsePredictive *Peripheral blood* •No relation between CD8^+^ T cell : Treg ratio and clinical response	(112, 113)
IL-6R Tocilizumab (RoActemra, 2009)	Pre-clinical *In vitro* CD4, CD25, CD127, FoxP3;CD8, CD25 *Animal model* CD4, CD25, FoxP3 Clinical *Peripheral blood* CD4, CD25, CD127, FoxP3;CD8, CD25, FoxP3	Pre-clinical *In vitro* •↑ % Tregs, followed by ↓ towards baseline level (probably apoptosis-related decline) •≈ % Tregs (HC) •≈ CD4^+^ Treg : CD8^+^ Treg ratio (HC)*Animal model* n.a. Clinical*Peripheral blood* •↑ number of Tregs •↑ or ↓ % Tregs •↑ % HLA-DR^+^ Tregs	Pre-clinical *In vitro* •↑ CD45RA^+^ Treg-mediated suppressive capacity (after expansion period, HC)*Animal model* •↑ Treg-mediated suppression (hM) •Restoration of Treg-mediated suppression (measured as ↑ body weight, hM)Clinical*Peripheral blood* •Treg-mediated suppressive capacity is not affected	Predictive *Peripheral blood* •↑ % Tregs is associated with clinical improvement or remission •↑ % (suppressive) CD39^+^ Tregs in responders compared to non-responders •Inverse correlation between % Tregs and disease activity •No association between % Tregs and changes in disease activity or clinical parameters •No association between Foxp3 mRNA : ROR-γt mRNA ratio and changes in disease activity	(114–129)
		•↑ % CD45RA^−^ Tregs •↑ % Helios^+^ Tregs •↓ % IL-17^+^ Tregs •↓ % FoxP3Δ2^+^ Tregs •↑ CTLA-4, CCR4 and Ki67 protein expression on/in Tregs •↑ FoxP3 mRNA expression in whole blood •↓ IL-10 mRNA expression in PBMCs •↑ Treg : activated effector CD4^+^ cell ratio •↑ Foxp3 mRNA : ROR-γt mRNA ratio in whole blood •↓ CD4^+^ IL-17^+^ cell : CD4^+^ CD25^high^ FoxP3^+^ cell ratio		•Inverse correlation between Treg : activated effector CD4^+^ cell ratio and disease activity •Positive correlation between Treg : effector CD4^+^ cell ratio and % STAT3^+^ cells •↓ CD4^+^ IL-17^+^ cell : CD4^+^ CD25^high^ FoxP3^+^ cell ratio is associated with reduced disease activity	
p40 subunit of IL-12 and IL-23 Ustekinumab (Stelara, 2009)	Pre-clinical n.a. Clinical *Peripheral blood* CD4, CD25, CD127, FoxP3	Pre-clinical n.a. Clinical *Peripheral blood* •≈ number of Tregs •↑ or ≈ % Tregs	Pre-clinical n.a. Clinical *Peripheral blood* •Treg-mediated suppressive capacity is not affected	n.a.	(130, 131)
VEGFR2 Ramucirumab (Cyramza, 2014)	Pre-clinical *In vitro* CD4, CD45RA, FoxP3 Clinical *Peripheral blood* CD4, CD45RA, FoxP3 *Tumour site* CD4, FoxP3; CD4, CD45RA, FoxP3	Pre-clinical *In vitro* •↓ % eTregsClinical*Peripheral blood* •≈ % eTregs*Tumour site* •↓ % eTregs (in TILs) •↓ % Ki67^+^ Tregs	n.a.	Prognostic *Tumour site* •↑ pre-treatment % eTregs (in TILs) is associated with partial response and longer progression-free survival	(132)

a *Summary of phenotypic markers used in the studies (in different combinations) to identify cells that are -according to the authors- regulatory cells*.

b *Study by Romano et al. (133) not taken into account, because of incorrect use of markers to determine Tregs (i.e., CD4^+^ CD25^+^ CD127^+^)*.

*↓, decreased level (compared to baseline or control); ↑, increase (compared to baseline or control); ≈, similar level (compared to baseline or control); C5, complement protein 5; CCR4, CC-chemokine receptor 4; CXCR4, CXC-chemokine receptor 4; eTreg, effector (activated) regulatory T cell; HC, healthy controls; HLA-DR, one of the human MHC class II molecules; hM, humanised mice; Ki67, intracellular marker for proliferation; n.a., not available; PBMC, peripheral blood mononuclear cell; TIL, tumour-infiltrating lymphocyte*.

#### Targets Not Directly Relevant for T Cells

Eleven mAb products were not expected to directly impact T cell function or survival and targets were therefore considered “non-relevant”. Published studies for mAb products targeting non-relevant molecules did indeed not report any Treg monitoring, except for belimumab and trastuzumab (Table 4). Belimumab targets the B lymphocyte stimulator (BLyS) protein, thereby blocking the activation of cells bearing the BLyS receptor. Target cells are primarily B cells, but also T follicular helper cells, which produce IL-21. Belimumab appears to reduce IL-21 production and subsequently restores Treg development at the expense of Th17 expansion (134).

**Table 4:** Overview of published studies for mAbs with a target not relevant for T cells.

***In vivo* target and monoclonal antibody (trade mark, year of EU registration)**	**Treg identification markers^**a**^**	**Effect of mAb on Treg frequency or phenotype**	**Effect of mAb on Treg functionality**	**Clinical associations**	**Key references**
BLyS (BAFF) Belimumab (Benlysta, 2011)	Pre-clinical *In vitro* CD4, CD25, CD127 Clinical *Peripheral blood* CD4, FoxP3	Pre-clinical n.a. Clinical *Peripheral blood* •↑ % Tregs •↑ Treg : Th17 cell ratio	Pre-clinical *In vitro* •Treg-mediated suppressive capacity is not affectedClinical n.a.	Predictive *Peripheral blood* •Inverse correlation between % Tregs and disease activity	(134)
Her2^b^ Ado-trastuzumab emtansine (Kadcyla, 2013)^c^	Pre-clinical *Animal model* CD4, CD25, FoxP3 Clinical *Peripheral blood* CD4, CD25, FoxP3 *Tumour site* FoxP3	Pre-clinical *Animal model* •↑ % Tregs (M) •↑ Ki67, CTLA-4 and T-bet protein expression in/on Tregs (M)Clinical*Peripheral blood* •≈ number of Tregs •≈ or ↓ % Tregs •↑ CD8^+^ T cell: Treg ratio •↓ Treg : Th17 cell ratio*Tumour site* •≈ or ↓ number of Tregs	n.a.	Predictive *Peripheral blood* •Negative correlation between mAb concentration and change in % Tregs •↑ % Tregs is associated with disease progression •↓ % Tregs is associated with progression-free survival *Tumour site* •↓ Number of Tregs in post-treatment tumour lesions is associated with clinical response	(135–142)

a *Summary of phenotypic markers used in the studies (in different combinations) to identify cells that are -according to the authors- regulatory cells*.

b *Study by Force et al. (143) not taken into account, because it was not clear which product (pertuzumab or trastuzumab) had effects on Tregs*.

c *For the clinical studies, is was not clear from the description whether trastuzumab (Herceptin) or ado-trastuzumab emtansine (Kadcyla) was used*.

*↓, decreased level (compared to baseline or control); ↑, increase (compared to baseline or control); ≈, similar level (compared to baseline or control); BLyS (BAFF), B lymphocyte stimulator (B cell activating factor); GD-2, glycolipid disialoganglioside-2; Ki67, intracellular marker for proliferation; M, mice; n.a., not available; PDGFR-α, platelet derived growth factor receptor-alpha; SLAMF7, signalling lymphocytic activation molecule F7*.

**Table 5:** Overview of mAbs for which no published scientific papers were available.

***In vivo* target group**	***In vivo* target**	**Monoclonal antibody (trade mark, year of EU registration)**
Target highly relevant for Tregs	PD-1	Cemiplimab (Libtayo, 2019)
Target relevant for the T cell population	EGFR	Necitumumab (Portrazza, 2016) Panitumumab (Vectibix, 2007)
	IL-1β	Canakinumab (Ilaris, 2009)
	IL-4R and IL-13R	Dupilumab (Dupixent, 2019)
	IL-5	Benralizumab (Fasenra, 2018) Reslizumab (Cinqaero, 2016) Mepolizumab (Nucala, 2015)
	IL-6	Sarilumab (Kevzara, 2017) Siltuximab (Sylvant, 2014)
	IL-17A	Ixekizumab (Taltz, 2016) Secukinumab (Cosentyx, 2015)
	IL-17RA	Brodalumab (Kyntheum, 2018)
	IL-23	Risankizumab (Skyrizi, 2019) Tildrakizumab (Ilumetri, 2018) Guselkumab (Tremfya, 2017)
	PD-L1	Durvalumab (Imfinzi, 2018) Atezolizumab (Tecentriq, 2017) Avelumab (Bavencio, 2017)
	TNF-α	Certolizumab pegol (Cimzia, 2009) Golimumab (Simponi, 2009)
Target not relevant for T cells	CD20	Ocrelizumab (Ocrevus, 2018) Obinutumumab (Gazyvaro, 2014) Ofatumumab (Arzerra, 2010)*
	CD22	Inotuzumab ozogamicin (Besponsa, 2017)
	CD33	Gemtuzumab ozogamicin (Mylotarg, 2018)
	GD-2	Dinutuximab (Unituxin, 2015)*
	Her2	Pertuzumab (Perjeta, 2013)
	PDGFR-α	Olaratumab (Lartruvo, 2016)*
	SLAMF7	Elotuzumab (Empliciti, 2016)

* *This mAb is now withdrawn from use in the European Union*.

Trastuzumab is indicated for Her2^+^ breast cancer and does not directly target the immune system. Nevertheless, Treg frequency and phenotype and their association with clinical outcome were evaluated both in human patients and in mice. One reason for assessing Tregs in breast cancer patients and the effect of trastuzumab on these cells may be that disease progression appears to be related to tumour-associated immunosuppression and FoxP3^+^ cell infiltration (135, 145–147). Indirect effects of the mAb on the balance between pro- and anti-inflammatory immune cells could therefore contribute to a more effective anti-tumour response.

For mAbs with a target outside the T cell population, still the number of the total T population or CD4^+^ and CD8^+^ cells were monitored, although no Treg monitoring was performed (Table 1), which is in line with the published reports on these products.

Taken together, we found that the depth of Treg (and T cell subset) immunomonitoring differs between products, depending on the likeliness that the mAb affects T cell functionality or survival. In addition, the extent of Treg evaluation varies between registration dossiers and published studies for individual mAbs. This is most probably because the majority of literature studies were academia-driven and were published only after marketing authorisation. Nevertheless, the involvement of the company in approximately half of these studies reveals that collaboration between industry and academia contributes to increased insight in treatment-related effects on the immune system.

### Other Products Affecting Treg-Relevant Targets

We took a pragmatic approach by evaluating mAb products EU-registered in a time period of 13 years without selecting for products that were actually meant to modulate the immune system. Only seven (of the 46 evaluated) products targeted molecules with high relevance for Treg function and survival. To determine whether other drug products with a Treg-relevant target took these suppressor cells into account in (pre-)clinical studies, we investigating two recently EU-registered Janus kinase (JAK) inhibitors indicated for rheumatoid arthritis. The JAK/STAT (i.e., signal transducer and activator of transcription) pathway is known to play an important role in the activation and survival of immune cells (148). Especially STAT5, a downstream target of the IL-2 receptor, is crucial for FoxP3 induction and Treg differentiation in the thymus (149, 150).

In both registration dossiers, effects of JAK inhibitors on lymphocyte and T cell subsets (cell count, phenotypic markers, cytokine production, and STAT phosphorylation) were determined, both clinically and pre-clinically. Clinically, also effects on Tregs (i.e., frequency) were investigated and a potential association between Tregs and the clinical response was explored. However, the amount of data presented in published studies (i.e., on local and systemic Treg frequency and functionality in mice and men, but also *in vitro*) was much more extensive than present in the dossier reports, although for one of the JAK inhibitors only one literature study was available (151–158).

## Discussion

### Study Limitations

Some limitations in our study need mentioning. First, we specifically selected EU-authorised mAb products (although not restricted to their registered indications). Therapies that did not reach the market or were still under review were not evaluated, although some of these products may target Tregs and thus dossiers could contain valuable information [e.g., isatuximab (159)]. In addition, we acknowledge that several mAb products with direct immune-related or even T cell-related targets are in the late-stage pipeline of several companies (160). Future MAAs containing data on Treg frequency or even functionality may thus be expected.

Second, mAb products authorised before 2006 were excluded, because the main increase in attention for and knowledge about Tregs occurred in the last decade. For several of these older immunomodulatory mAb products with a direct impact on the T cell population (e.g., infliximab, adalimumab, alemtuzumab, daclizumab), recently published immunomonitoring studies involved Treg frequency and functionality, because of their importance in the disease pathophysiology and to (further) elucidate the pharmacological mechanisms of action in lack-of-response issues or for biomarker definition (161, 162). Tregs are also monitored for old mAbs targeting non-T cell receptors, such as CD20 (rituximab) (163, 164). Above-mentioned studies investigating effects of recently authorised products or relatively old products on the immune system could contribute to our knowledge of T cell subsets.

Third, mAb products indicated for infections (e.g., caused by *Clostridium difficile* or HIV) or immune-related diseases with a distinct pathophysiology (such as paroxysmal nocturnal haemoglobinuria) were also excluded. Only a few mAb products are registered for these indications and it is expected that data concerning Tregs in existing dossiers will be limited.

Fourth, for literature reports, we limited our search to the same products as described for MAA dossiers and thus excluding studies with non-registered human or “mousenised” mAbs against the same target. We acknowledge that these excluded studies would be helpful when more insight in efficacy or safety-related effects of mAb products on specific immune cells would be required. Nevertheless, to establish the relevance of experience with such non-registered products, interpretation of the published data would be needed, which was not the aim of our study.

### Value of Treg Monitoring

Tregs have a crucial function in regulating immune responses to dampen inflammation, limit tissue damage and prevent auto-reactivity. Pharmacological impact on their number and/or (local) activity, either directly or indirectly, is likely to contribute to (or impair) clinical responses or to adverse events. Therefore, monitoring effects of immunomodulatory products on T cells -including Tregs- should be part of (pre-)clinical studies.

In addition, Tregs or specific Treg subsets may turn out to be predictive biomarkers for specific diseases or patient populations. We noted that in the majority of mAb product dossiers, no clinical relevance was estimated for treatment-induced changes in Treg frequency or phenotype. For only two products, an association was determined between Treg frequency and the clinical response. Published reports, on the other hand, frequently mentioned associations between the amount of (local) Tregs and clinical outcome. Thereby, Tregs could act as biomarker for the clinical response (144). Associations with the baseline Treg level prior to treatment may be used as *prognostic* biomarker, for example to select patients eligible for mAb (anti-tumour) therapy. Changes in Treg level following treatment may act as *predictive* marker of the mAb-mediated clinical response, in auto-immune as well as in neoplastic indications. Nonetheless, interpretation of clinical associations and treatment-related Treg effects is still rather difficult. For example, differentiation between effects that are a direct consequence of the medicinal product activity or a result of disease remission is usually not accomplished. In addition, the clinical significance of fluctuations in Tregs during or after therapy remains to be established.

In general, immunomonitoring has a substantial value to assess the effectiveness and safety of therapeutic interventions and to select patients eligible to these treatments (2, 165). On the level of T cell subsets, scientific knowledge regarding immune responses is growing exponentially (also for older immunomodulatory treatments) and this knowledge should be taken into account when selecting for specific immunomonitoring parameters. But we consider that the general added value of measuring Tregs in (pre-)clinical studies is not yet sufficiently clear and their contribution to the clinical response requires more extensive analysis. This would include gathering information regarding the Treg role in disease pathophysiology and therapy-related adverse events. Regulatory authorities need this information to estimate the value of Tregs and the necessity to take treatment-related Treg changes along in the benefit-risk assessment. Tregs should therefore be taken into account as exploratory parameter in (pre-)clinical studies, either prior to MAA or post-registration in collaboration with academia.

Despite growing knowledge regarding treatment effects on Tregs, we observed a high variability in data between the different studies, probably due to heterogeneity of the experimental approach. Studies differed in markers used to identify Tregs, in methods to measure their functionality and in assay read-out techniques. Also tissues and species used to monitor Tregs, the time between treatment and analysis points varied between studies. Moreover, the treatment protocol (e.g., administration route, number and quantity of doses and dose intervals, concomitant therapies), therapeutic indication and the number of patients also added to study heterogeneity. D'Arena et al. ran into the same problem of heterogeneity when evaluating the relevance of Tregs as biomarker in the context of hematologic malignancies. Their study also exemplified “the need for more standardised approaches in the study of Tregs” (166). Thus, harmonisation of Treg identification and monitoring is required before these cells can become actual endpoints in clinical investigations or can be used as prognostic or predictive biomarker (25, 144).

Apart from this lack of harmonisation, the scientific knowledge is too limited to demand or guide Treg monitoring in registration dossiers. Nevertheless, we hereby stimulate companies (and academia) to take these cells into account in their investigations or to collaborate with academia to perform T cell subset-specific studies (post-registration).

### Recommendations for (Pre-)clinical Studies

We will end this review with some specific points-to-consider for Treg (and other T cell subset) monitoring in pre-clinical and clinical studies.

#### Sampling

Treg monitoring (both clinically and pre-clinically) could be restricted to products with a target known to play a vital role in T cell development, differentiation, functionality, or survival. Especially when the target is related to regulation of the immune system and loss-of-function would considerably increase the risk of auto-immunity [e.g., CTLA-4 expression on Tregs (27)], monitoring the frequency and functionality of immune cells closely related to this target may significantly add to the identification of potential safety concerns early in product development. Obviously, for products containing (*ex vivo* expanded) Tregs or for therapies typically aiming to enhance Treg activity (e.g., tolerogenic dendritic cells), analysis of Treg frequency and/or function will be imperative (24). For immunomodulatory treatments with a target in the non-T cell compartment, a risk-based approach could identify whether monitoring of T cell subset responses would be required to substantiate clinical data.

We suggest to add several Treg-related markers to an existing immune monitoring panel (see below in subsection Identifying Tregs). When this would not be feasible, one could retain clinical samples to be able to retrospectively measure effects on specific T cell or Treg subsets when required [as recently performed for unexpected events with nivolumab (167–169)]. More “standard” Treg monitoring could then be restricted to pre-clinical investigation.

What samples would be most appropriate? In general, in humans peripheral blood is the most accessible compartment for multiple analyses over time. Nevertheless, changes in circulating T cell subsets may not accurately reflect the local environment. Furthermore, it has been reported that the ratio and phenotype of Treg subsets at tumour sites differ substantially from peripheral blood (8, 9, 25, 132, 170). Therefore, when feasible, treatment effects on local T cell subsets may be taken into account as well (26).

We noted that pre-clinical *in vitro* pharmacologic studies are frequently performed with cells from healthy donors. This can be acceptable, but using cells from patients may have added value when the disease has impaired the intrinsic function of the cells. For example, patients with giant cell arteritis can have a defect in their FoxP3 protein, which affects the suppressive capacity of the Tregs, but could be pharmacologically corrected (114).

#### Identifying Tregs

In general, treatment-related effects on the immune system are dose-dependent and difficult to predict. For example, lymphocyte-depleting approaches (such as anti-thymocyte globulin) do not simply deplete all T cells, but also act as immunosuppressant by, for example, converting effector into regulatory T cells and by preserving or even expanding already existing Tregs. An increased Treg to conventional T cell ratio may therefore be an unexpected effect of T cell-depleting antibodies (21, 36, 171, 172). This indicates that monitoring drug-mediated effects on the whole T cell population may not correctly predict effects on T cell subsets. Therefore, these analyses should preferably discriminate between effector and regulatory T cells, at least for products indicated to specifically target T cells.

Accurately defining Tregs is, however, a challenge. Although there are several useful reviews available that highlight different markers and cytokines that may help identifying Tregs, there is no unique Treg marker (7, 22, 24, 173–175). Expression of FoxP3, the master regulator of classical CD4^+^ Tregs, is not limited to human regulatory cells: effector T cells transiently upregulate FoxP3 expression after activation and also other immune cells and even tumour cells may express this transcription factor (176–181). In addition, not all regulatory T cell subtypes express FoxP3. Moreover, FoxP3 cannot be used to isolate Tregs alive for *ex vivo* functionality testing. Combined use of several (surface) markers will therefore be needed to identify and purify Tregs. In contrast to murine CD4^+^ CD25^+^ regulatory T cells, only CD4^+^ cells with a high level of CD25 expression have a suppressive capacity in humans. The other CD4^+^ CD25^+^ T cells are activated effector T cells. According to the vision of several experts in the field, CD3, CD4, CD25, CD127, and FoxP3 are the minimally required markers to define human Treg cells in flow cytometric samples and addition of Ki67 and CD45RA/RO could provide information on the activation status of Tregs (173) or improve selection of pure Treg fractions (182). Such Treg panel would also allow for monitoring of different effector T cell subsets (naïve/memory state of both CD4^+^ and CD8^+^ T cells). The experts also emphasised that a proper flow cytometric gating strategy will improve the reliability and purity of the defined Treg population, and in the meantime diminish inter-assay variability (173, 183). Recently, Pitoiset et al. provided a standardised protocol to monitor Tregs in multicentre clinical trials, using above-mentioned markers (184).

One should, however, keep in mind that there are phenotypic differences between circulating Tregs and Tregs at sites of inflammation (173). A more precise discrimination of Treg subtypes may thus be needed, especially in the peripherally-induced (heterogeneous) population (25, 185). And differentiating between naïve and activated Treg subsets may be needed to find treatment-related effects or clinical associations (186, 187). Nevertheless, distinguishing between Treg subsets (especially thymus- vs. periphery-derived cells) is rather challenging (3–5, 188–190). Measuring the amount of demethylation of the FoxP3 gene may provide insight in the stability of FoxP3 expression and thereby distinguish thymus-derived Tregs from peripherally-induced Tregs and activated conventional T cells (191, 192). Nevertheless, this demethylation status analysis requires a highly pure lymphocyte sample.

#### Functionality Testing of Tregs

There are various methods to analyse the suppressive capacity of Tregs. Most of these assays aim to measure inhibition of effector T cell proliferation or cytokine production, although cytotoxicity inhibition may also be used as read-out (193, 194). The requirement of a rather large amount of autologous cells for such co-cultures would make these types of assays less suitable for patient samples (195). Indeed, we found in several registration dossiers that such testing was considered clinically, but impossible to perform. In addition, the *in vivo* functionality may be impacted by the tissue environment, which is difficult to mimic *in vitro* (26). Moreover, impaired *in vivo* suppressive function is not always reflected by results from an *in vitro* assay (20).

Treg functionality was only (pre-clinically) analysed in one of the 46 mAb dossiers evaluated. Lack of functionality testing is probably the result of difficulties with assay design. Nevertheless, there are literature examples where Treg functionality testing appeared possible (62). We therefore would like to draw attention to different approaches that may enhance the possibility of Treg function analysis. Instead of using autologous cells, a mixed lymphocyte reaction may be considered (196, 197). In addition, to prevent long co-culture periods, a surrogate read-out (i.e., inhibition of activation marker expression instead of proliferation) could be used (198–200). Identification of functional Tregs via marker gene analysis (e.g., FoxP3, CTLA-4, and IL-10) may also be a simple and quick method, although the level of mRNA expression does not necessarily reflect protein expression and this read-out is also considered surrogate for Treg functionality (201). Simply distinguishing between resting and activated Tregs and effector T cells can also provide information about the presence or absence of suppressive T cells in a sample (182, 187). More considerations and technical challenges for Treg functionality assays can be found in the public domain (25, 115, 183, 194, 202–204).

### Future Perspective and Conclusion

We are now starting to understand the role of different T cell subsets in disease pathogenesis and immunotherapeutic mechanisms of action. This provides the opportunity to selectively target specific subpopulations rather than a whole T cell population to improve the effectiveness and safety of immunomodulatory therapies. In addition, monitoring the activation status, function and amount of specific T cell subsets could assist in identifying the patients that would most likely benefit from therapy (2). A risk-based approach is considered helpful to select products that would require T cell subset monitoring to more reliably assess the product's benefits and risks.

Immunomonitoring, as proposed in this review, will also help to enrich our knowledge about Tregs and their association with the clinical response. This will, however, require accurate phenotypic identification of regulatory subsets and further investigation of the clinical relevance of treatment-induced changes in their levels. To obtain and report such information in a systematic way, a collaboration between industry and academia will be required (205).

We believe that there are still many issues to address before Tregs can be used as biomarkers for targeted therapies, but gathering knowledge about Treg subpopulations in health and disease will eventually shed more light on the (pre-)clinical value of these regulatory cells. This will ultimately result in more concrete regulatory guidance for T cell (and particularly Treg) monitoring in studies used for marketing authorisation.

## Data Availability Statement

The datasets generated for this study will not be made publicly available because they contain confidential information from registration dossiers. Requests to access the datasets should be directed to mh.hoefnagel@cbg-meb.nl.

## Author Contributions

AW collected the data and wrote the first draft of the manuscript. AW and MH were involved in the interpretation of the data. All authors contributed to the conception and design of the work, manuscript revision, read, and approved the final version.

### Conflict of Interest

The authors declare that the research was conducted in the absence of any commercial or financial relationships that could be construed as a potential conflict of interest.
